# The effect of lyophilized dried cornelian cherry (*Cornus mas* L.) intake on anthropometric and biochemical parameters in women with insulin resistance: A randomized controlled trial

**DOI:** 10.1002/fsn3.3725

**Published:** 2023-10-08

**Authors:** Zehra Margot Celık, Mehmet Sargin, Havva Gonca Tamer, Fatma Esra Gunes

**Affiliations:** ^1^ Department of Nutrition and Dietetics Marmara University Faculty of Health Sciences Istanbul Turkey; ^2^ Faculty of Medicine, Family Medicine Istanbul Medeniyet University Istanbul Turkey; ^3^ Faculty of Medicine, Internal Medicine Istanbul Medeniyet University Istanbul Turkey; ^4^ Department of Nutrition and Dietetics, Faculty of Health Sciences Istanbul Medeniyet University Istanbul Turkey

**Keywords:** cornelian cherry, *Cornus mas*, insulin resistance, medical nutrition therapy, nutritional status

## Abstract

The aim of this study was to determine the effect on the anthropometric and biochemical parameters for women with insulin resistance when lyophilized dried cornelian cherry (*Cornus mas* L., CM) was added to medical nutrition therapy (MNT). The study was conducted with 84 women aged 18–45, who had been diagnosed with insulin resistance. Participants were randomized into four groups: MNT + 20 g lyophilized dried CM group (DCm, *n* = 22), MNT group (D, *n* = 21), only 20 g lyophilized dried CM group (Cm, *n* = 21), and the control group (C, *n* = 20). All participants were followed for 12 weeks. While pre‐ and post‐intervention biochemical parameters were recorded from patient files, anthropometric measurements and food consumption records were taken every 15 days. Pre‐intervention groups were homogeneously distributed. Post‐intervention, among the groups, all anthropometric measurements were similar between the DCm and D, while the percentage of decrease in insulin resistance‐related parameters was approximately two times greater in DCm than in D (*p* < .05). When the Cm and C were compared, it was found that all post‐intervention anthropometric measurements were similar, but the percentage of decrease in fasting blood glucose, fasting insulin, and HOMA‐IR (Homeostasis Model Assessment‐Insulin Resistance) values were greater in C (*p* < .05). In this study, it was concluded that CM consumption resulted with a decrease in insulin resistance‐related biochemical parameters independent of body weight change. Nevertheless, MNT has positive effects on women with insulin resistance, and adding lyophilized dried CM to MNT improves insulin resistance‐related parameters and may be beneficial for preventing the development of diabetes.

## Graphical abstract

Adding 20 g lyophilized dried cornelian cherry (*cornus mas* L.) to a medical nutrition therapy, reduced fasting insulin, c‐peptide and HOMA‐IR levels nearly twice as much (%) than only medical nutrition therapy.

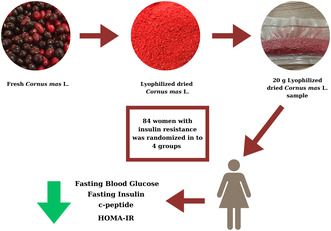

## 1 INTRODUCTION

Cornelian cherry (*Cornus mas* L.; CM) is rich in ascorbic acid and phenolic compounds, in particular anthocyanin, which gives the fruit its color (Didin et al., 2000; Rudrapaul et al., 2015). Even though it has been found that different parts of CM contain approximately 10 anthocyanins as well as many different phytochemicals, the predominant anthocyanin groups in CM are cyanidin 3‐O‐galactoside and pelargonidin 3‐O‐galactoside (Dinda et al., 2016; Jayaprakasam et al., 2006; Pawlowska et al., 2010). There are studies in the literature on how the use of polyphenols improves insulin resistance, prevention, and treatment of type 2 diabetes mellitus (T2DM) (Cheng et al., 2014; Kappel et al., 2013; Rojo et al., 2012; Yan & Zeng, 2017). In a meta‐analysis, the use of routine dietary flavonoids to help prevent T2DM development was evaluated, and as for dose–response analysis, it was found that there was a 5% decrease in T2DM risk for every increase of 300 mg/day in total flavonoid intake (Xu et al., 2018). Anthocyanins have been shown to reduce peripheral blood glucose levels and insulin resistance in both diabetic and obese animal models and cross‐sectional human studies (Jayaprakasam et al., 2005; Sarikaphuti et al., 2013). Research shows that CM, rich in anthocyanins, inhibits α‐glucosidase enzyme activity and this enzyme appears to prevent the hydrolysis of carbohydrates and thus lower blood glucose levels (Matsui et al., 2001). Anthocyanin‐rich CM also has an activating effect on G‐protein receptors that improve insulin action through the receptors that cause glucose reduction (Asgary et al., 2014). In addition, it is thought that the known anti‐inflammatory effects of anthocyanins may have potential benefits in improving insulin resistance caused by low‐grade chronic inflammation (Belwal et al., 2017; Imai et al., 2004).

Obesity, insulin resistance, and T2DM, which are increasing day by day in the world and Turkey, are becoming a global problem. According to Turkey Nutrition and Health Survey (TBSA) 2019 data, the obesity rate in women (31.1%) is higher than men (23.8%). In the Turkish Diabetes Epidemiology II (TURDEP‐II) study conducted in Turkey, it was found that the prevalence of T2DM in the general population aged 20 years and older was 13.7% and the rate in women was higher than men (Satman et al., 2013). In a study conducted with 2013 participants in Turkey, the prevalence of insulin resistance was found to be higher in women (35.6%) compared to men (30.1%) (Demir et al., 2020). The most effective treatment for insulin resistance is reducing body weight with MNT and by increasing physical activity (Sievenpiper et al., 2002). Evidence suggests that there are no standard values for an ideal percentage of energy from carbohydrates, protein, and fat for the treatment of insulin resistance. Therefore, the distribution of macronutrients in MNT should be based on an individualized assessment of current eating habits, preferences, and metabolic goals (Evert et al., 2019). In addition to macronutrients, it has been revealed that micronutrients in the diet have specific positive effects (polyphenols, vitamins, minerals, etc.) on the treatment of insulin resistance (Cheng et al., 2014; Choi et al., 2005; Kayaniyil et al., 2010, 2011).

It has been observed that in the literature, studies investigating the effects of anthocyanins on insulin resistance, fasting blood sugar, and diabetes mostly use extracts, and the information on the effects of eating the fruit itself is quite limited. This study was planned and conducted to determine the effect of adding lyophilized dried cornelian cherry (*Cornus mas* L.) rich in polyphenols to the medical nutrition therapy of women with insulin resistance.

## 2 MATERIALS AND METHODS

### 2.1 Study design

For this randomized controlled trial (RCT), insulin resistance was calculated using the HOMA‐IR (Homeostasis Model Assessment‐Insulin Resistance), taking the fasting insulin and fasting blood glucose values from the patients' files according to blood tests performed 15 days prior to the trial. Patients with a HOMA‐IR value of 2.5 and above, which is the cut‐off point for insulin resistance, were included in the study (Ascaso et al., 2003).

Women who met the inclusion criteria were divided into four groups by block randomization method. Patients were divided into Diet + *Cornus mas* L. group (DCm), where 20 g/day of lyophilized dried cornelian cherry was given in addition to the MNT program; diet group (D), which received only the MNT program; *Cornus mas* L. group (Cm), which was given 20 g/day of lyophilized dried cornelian cherry; and control group (C), which did not receive any intervention.

The study was approved by the Marmara University Faculty of Medicine Clinical Research Ethics Committee (09.2018.652) and was carried out at the obesity polyclinic of the endocrinology department of a tertiary hospital in Istanbul/Turkey between May 2021 and April 2022, in accordance with the Helsinki declaration.

### 2.2 Participants

This study was conducted with women aged 18–45 years who applied to the obesity outpatient clinic and were diagnosed with insulin resistance by a physician. Exclusion criteria from the study were state of menopause, being on hormone therapy, using endocrine‐related (antidiabetic, thyroid hormone regulator, etc.) or immune system‐modulating drugs, being pregnant or lactating, having any chronic disease other than insulin resistance, recent major surgery, or history of cancer, allergy, or intolerance to cornelian cherry. Those who were diagnosed with COVID‐19 during the study were also excluded from the study. All participants signed informed consent form before taking part in the study.

### 2.3 Preparation of the *Cornus mas L.* packages

In August–September 2020, cornelian cherries grown in Erzurum, Uzundere Gölbaşı Neighborhood, were purchased. The collected *Cornus mas* L. samples were identified by Dr. Gizem Emre from Marmara University Faculty of Pharmacy (Istanbul, Turkey) (Voucher Number: MARE‐22458). Fresh CM fruits purchased for the study were mashed with a 32‐mm‐pore pulper machine to remove the seeds; the outer skin of the CM was included in the puree. Afterward, the lyophilized drying process was carried out by sublimation with a G‐Ray 125 freeze‐dry machine.

The lyophilized dried powder CM was analyzed to determine the amount to be given to the participants. The carbohydrate profile was analyzed using high‐performance liquid chromatography (HPLC) with refractive index detector (RID) (Agilent 1100) and Hypersil APS‐2 (5 μm, 250 × 4.6 mm) column.

For the total anthocyanin analysis, the content of the extracted samples was determined according to the method reported by Giusti and Wrolstad (2001), which is based on the work of Fuleki and Francis (1986). The total amount of monomeric anthocyanin was calculated in terms of cyanidin 3‐glucoside.

In this study, 20 g lyophilized dried powder CM containing 11.94 g carbohydrates and 237.55 mg anthocyanins was used. Lyophilized dried powder CM was weighed with a scale that had precision to within 1 g and was then packed and vacuumed per 20 g. The 15 days CM packages were prepared weekly and delivered to the participants during their polyclinic visit.

### 2.4 Trial intervention

After the participants were randomly placed in the study groups, they were invited to the polyclinic every 15 days for a period of 12 weeks. In the control visits, CM‐consuming groups were given lyophilized dried powder CM packages to consume for 15 days. They were asked to consume it only mixed with water or as plain powder. For all participants, 3 days food consumption records and anthropometric measurements were taken. Routine biochemical parameters were recorded in their files at the end of the 12 weeks intervention period.

To provide similar planning for the participants, a standard MNT form on which quantity changes could be made was used. The content of the MNT was calculated individually as 45%–55% carbohydrates, 15%–20% protein, and 25%–30% dietary fat (Evert et al., 2019). The fruit intake of all participants was set the same, as two portions per day. In addition, it was arranged that 75% of the carbohydrate content of the planned MNT programs consisted of complex carbohydrates.

In this study, conducted with CM that contains high anthocyanin, consumption of red–purple fruits with high anthocyanin content was eliminated for all groups in order not to affect the results. In group D, two portions of fruit were left to the preference of the participants. For DCm, one portion of fruit was left to the preference of the participant, and the second fruit portion was the 20 g CM package.

### 2.5 Data collection and evaluation

The data of the study were collected via the face‐to‐face interview method with a questionnaire form that had been prepared and structured by the researchers. The questionnaire consisted of questions about demographic information, health status, and physical activity habits.

### 2.6 Biochemical parameters and anthropometric measurements

Patients who apply to the obesity polyclinic of the endocrinology department are given routine laboratory blood tests every 12 weeks. After obtaining the necessary permits, all biochemical parameters at the baseline and the end of the 12 weeks intervention were recorded from the routine laboratory blood test results.

The anthropometric measurements taken in the study were height (cm) (accuracy ±0.1 cm), body weight (kg) (accuracy ±0.1 kg), body mass index (BMI; kg/m2), waist circumference (WC; cm), hip circumference (HC; cm), mid‐upper arm circumference (MUAC; cm), neck circumference (NC; cm), and body fat percentage (BFP; %). For the measurements, according to the standards, a stadiometer, a non‐flexible tape measure, and a bioelectrical impedance analyzer (BIA) device (Tanita MC 780 MA) were used. All anthropometric measurements were carried out by the researchers at the outpatient clinic.

Body mass index (BMI) was calculated as body weight divided by the square meter of height [body weight (kg)/height (m)^2^] and classified according to WHO classification (WHO, 2023).

### 2.7 Food consumption records

The food consumption status of the participants were evaluated using a 3 days food consumption record. It was requested that the days to be recorded be 2 consecutive weekdays and 1 day at the weekend. The form was given each time the participants visited the polyclinic, and they were asked to fill it out and bring it to the next visit. For it to be filled in correctly, a short verbal training, including portion sizes, was given to the participants by the researchers. The Nutrition Information System (BeBis) 9.0 Full Version computer program was used to evaluate the energy and macro‐ and micronutrient intakes.

### 2.8 International physical activity questionnaire–short form (IPAQ‐SF)

The 7‐item International Physical Activity Questionnaire–Short Form (IPAQ‐SF) was used to evaluate the physical activity levels of the participants (Craig et al., 2003). The validity and reliability study of this questionnaire for Turkish society was conducted by Öztürk (2005). Physical activity scores were determined for each part of the activity level and then calculated as individual metabolic equivalent activity (MET). While classifying, scores below 600 were grouped as “inactive,” scores between 600 and 3000 as “minimal active,” and scores over 3000 as “active” (Physical Activity Guideline, 2023).

### 2.9 Data analysis and statistics

The data obtained were statistically evaluated with the SPSS 24.0 package program. The compliance of the variables with normal distribution was checked with the one‐sample Kolmogorov–Smirnov test. For continuous data, the median (25th quartile–75th quartile) was given. The delta (Δ) values [Delta = (new – old)/old x 100] were calculated for the percent differences between pre‐intervention and post‐intervention parameters. The Kruskal–Wallis test was used to compare the median values of data from more than two groups that did not fit the normal distribution, and the Mann–Whitney *U* test was used to determine which groups caused the difference in significant data. The Wilcoxon test was used to compare the data of pre‐ and post‐intervention. All analyses were calculated at 95% confidence interval and statistical significance was accepted as *p* < .05.

## 3 RESULTS

**FIGURE 1:**
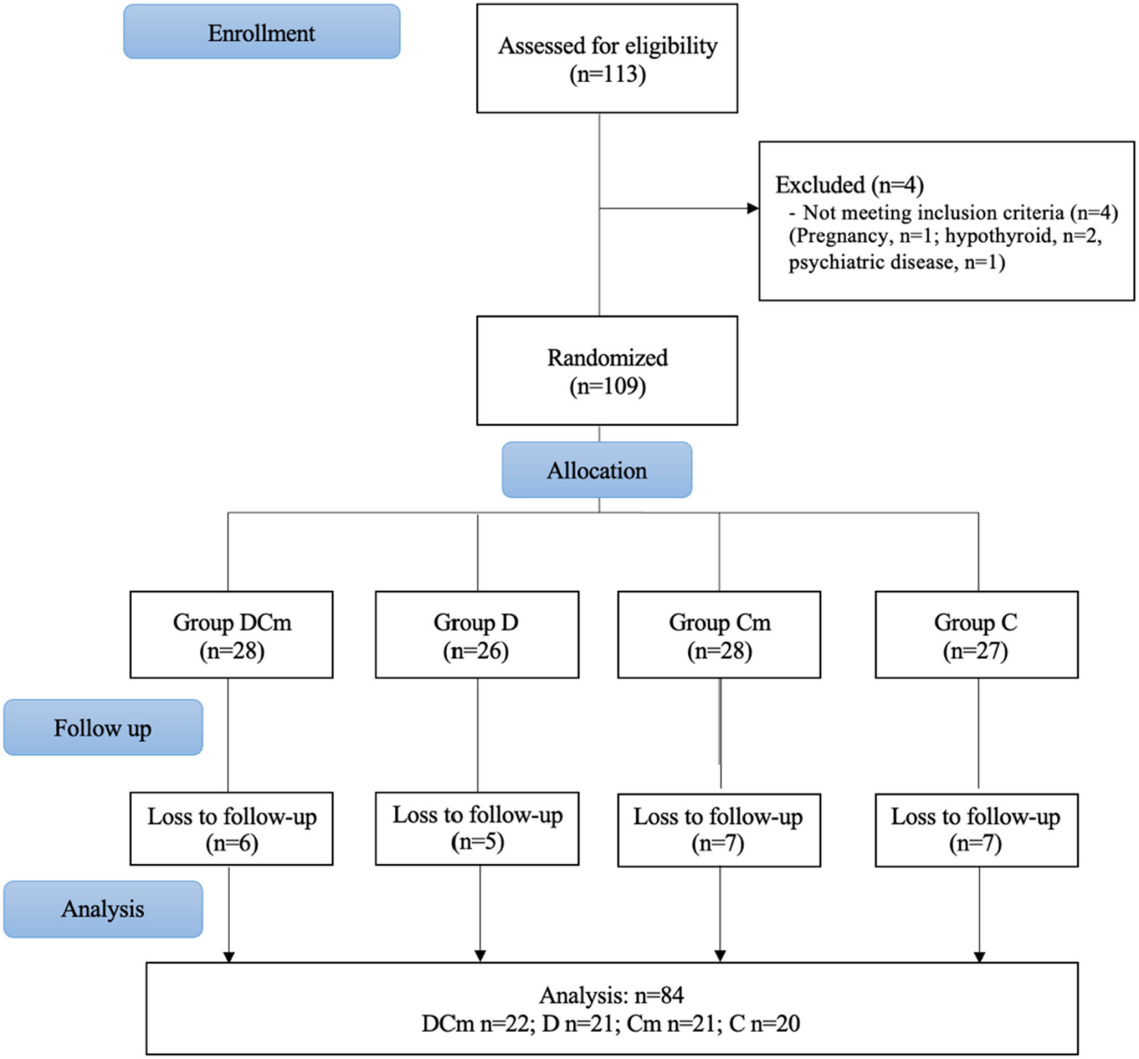
CONSORT flow chart. C, Control group; Cm, *Cornus mas L*. group; D, Diet group; DCm, Diet + *Cornus mas L*. group.

One hundred thirteen women who applied to the outpatient clinic, who met the research criteria, and who agreed to participate in the study were included. After 29 participants abandoned the study for several reasons, including being diagnosed with COVID‐19, staying in isolation due to a family member being diagnosed with COVID‐19, or voluntarily dropping out of the study due to concerns about coming to the hospital and the threat of contracting COVID‐19, the study was completed with 84 participants (Figure 1).

The baseline demographic characteristics of the participants are presented in Table 1. As shown, the groups had a similar statistical distribution for all parameters before the intervention. Median age of the participants was found to be 35.0 (24.0–41.0) years. It was determined as 38.0 (26.8–41.3) years for DCm, 36.0 (23.0–43.0) years for D, 29.0 (24.0–41.5) years for Cm, and 29.5 (22.0–40.8) years for C, and the medians of the groups were found to be similar (*p* = .455) (not shown in table).

**TABLE 1:** Baseline demographic characteristics.

	All (*n* = 84) *n* (%)	DCm (*n* = 22) *n* (%)	D (*n* = 21) *n* (%)	Cm (*n* = 21) *n* (%)	C (*n* = 20) *n* (%)	*p*
Education status						
Primary school	16 (19.0)	3 (13.6)	7 (33.3)	3 (14.3)	3 (15.0)	.092
Middle school	7 (8.3)	2 (9.1)	2 (9.5)	1 (4.8)	2 (10.0)	
High school	37 (44.0)	6 (27.3)	9 (42.9)	9 (42.8)	13 (65.0)	
University and above	24 (28.6)	11 (50.0)	3 (14.3)	8 (38.1)	2 (10.0)	
Working status						
Employed	18 (21.4)	7 (31.8)	3 (14.3)	3 (14.3)	2 (25.0)	.418
Unemployed	66 (78.6)	15 (68.2)	18 (85.7)	18 (85.7)	15 (75.0)	
Marital status						
Married	52 (61.9)	16 (72.7)	14 (66.7)	12 (57.1)	10 (50.0)	.440
Single	32 (38.1)	6 (27.3)	7 (33.3)	9 (42.9)	10 (50.0)	
Regular exercise						
Yes	29 (34.5)	6 (27.3)	8 (38.1)	8 (38.1)	7 (35.0)	.861
No	55 (65.5)	16 (72.7)	13 (61.9)	13 (61.9)	13 (65.0)	
Physical Activity Level						
Inactive	59 (70.2)	16 (72.7)	14 (66.7)	13 (61.9)	16 (80.0)	.614
Minimal active	25 (29.8)	6 (27.3)	7 (33.3)	8 (38.1)	4 (20.0)	
Active	0 (0.0)	–	–	–	–	

Abbreviations: C, Control group; Cm, *Cornus mas L*. group; D, Diet group; DCm, Diet + *Cornus mas L*. group.

Data are presented as *n* (%).

*p* values were calculated using chi‐square test.

Table 2 comparatively shows the effects of interventions on anthropometric measurements. As seen, following an MNT program significantly reduced all anthropometric measurements from baseline in DCm and D. The groups that did not receive an MNT program (Cm and C) had no statistically significant change regarding body weight, BMI, and BFP. It has been demonstrated that there was no statistically significant difference in the anthropometric measurements between the groups at the end of the intervention; however, when the Δchange was calculated for the groups, it was found that the change in measurements was significantly greater in DCm and D compared to Cm and C.

**TABLE 2:** Changes in anthropometric measurements from baseline to after intervention.

Parameters	DCm (*n* = 22)	D (*n* = 21)	Cm (*n* = 21)	C (*n* = 20)	*p* ^1^
Body weight (kg)					
Baseline	89.0 (84.5 – 97.1)	85.2 (80.3 – 104.7)	86.5 (80.1 – 104.6)	88.6 (77.6 – 99.1)	.932
After intervention	85.3 (76.1 – 91.3)	80.9 (74.2 – 94.3)	86.4 (79.1 – 105.3)	89.8 (75.8 – 98.1)	.363
*p* ^2^	**<.001**	**<.001**	.088	.411	
Δchange	−6.73^**a**^ (−7.85 – −4.50)	−5.41^**a**^ (−7.72 – −4.32)	−0.84^**b**^ (−2.64 – 0.87)	−0.46^**b**^ (−2.34 – 0.97)	**<.001**
Body mass index (kg/m2)					
Baseline	33.5 (30.5 – 36.6)	32.7 (31.0 – 37.6)	34.3 (29.3 – 39.6)	32.5 (30.8 – 38.4)	.952
After intervention	31.4 (27.1 – 34.0)	31.1 (29.2 – 34.6)	33.8 (28.4 – 39.1)	33.1 (30.0 – 37.6)	.274
*p* ^2^	**<.001**	**<.001**	.095	.421	
Δchange	‐6.67^**a**^ (−7.63 – −4.37)	‐5.81^**a**^ (−7.65 – −4.38)	−1.02^**b**^ (−2.48 – 0.00)	−0.52^**b**^ (−2.40 – 1.04)	**<.001**
Waist circumference (cm)					
Baseline	99.0 (92.6 – 108.5)	101.0 (96.0 – 114.6)	103.0 (95.0 – 111.5)	103.8 (94.0 – 113.5)	0.706
After intervention	91.0 (85.8 – 101.5)	92.0 (87.5 – 106.5)	101.0 (93.5 – 107.5)	104.0 (93.3 – 111.0)	0.052
*p* ^2^	**<.001**	**<.001**	**<.001**	**.026**	
Δchange	−6.08^**a**^ (−7.73 – −4.69)	−6.91^**a**^ (−8.76 – −5.06)	−0.98^**b**^ (−4.09 – 0.00)	−1.27^**b**^ (−3.70 – 0.00)	**<.001**
Hip circumference (cm)					
Baseline	122.5 (113.8 – 129.8)	116.0 (113.5 – 131.0)	121.0 (113.0 – 129.0)	122.0 (113.0 – 130.0)	.986
After intervention	116.0 (105.5 – 123.5)	112.0 (109.0 – 119.5)	119.0 (109.5 – 126.0)	120.5 (112.0 – 128.0)	.272
*p* ^2^	**<.001**	**<.001**	**<.001**	**.020**	
Δchange	−5.04^**a**^ (−6.52 – −3.49)	−5.17^**a**^ (−6.74 – −4.24)	−2.46^**b**^ (−3.98 – −0.78)	‐0.78^**b**^ (−2.29 – 0.00)	**<.001**
Mid‐upper arm circumference (cm)					
Baseline	34.3 (32.0 – 36.0)	34.0 (32.5 – 36.5)	34.0 (31.0 – 38.5)	33.0 (30.3 – 35.8)	.664
After intervention	32.0 (30.0 – 33.3)	32.0 (30.0 – 34.0)	33.5 (30.0 – 37.0)	33.0 (29.5 – 35.0)	.441
*p* ^2^	**<.001**	**<.001**	**<.001**	**.009**	
Δchange	−5.80^**a**^ (−8.57 – −3.08)	−7.89^**a**^ (−8.96 – −5.64)	−2.63^**b**^ (−4.17 – 0.00)	−2.86^**b**^ (−3.33 – 0.00)	**<.001**
Neck circumference (cm)					
Baseline	36.0 (34.9 – 39.3)	37.0 (35.8 – 39.5)	37.0 (35.5 – 40.5)	36.0 (34.1 – 39.8)	.591
After intervention	34.5 (33.8 – 36.5)	36.0 (35.0 – 38.5)	36.0 (35.0 – 39.5)	36.0 (34.0 – 39.0)	.204
*p* ^2^	**<.001**	**<.001**	**.002**	**.034**	
Δchange	−4.29^**a**^ (−5.44 – −2.94)	−2.86^**ab**^ (−5.73 – −2.47)	‐2.44^**b**^ (−4.82 – 0.00)	0.00^**b**^ (−2.65 – 0.00)	**<.001**
Body fat percentage (%)					
Baseline	37.6 (33.4 – 41.2)	38.5 (33.2 – 40.8)	38.0 (34.0 – 40.6)	37.6 (33.5 – 40.8)	.999
After intervention	36.9 (29.6 – 39.9)	35.1 (31.2 – 38.5)	37.6 (34.9 – 41.3)	38.5 (34.7 – 40.4)	.209
*p* ^2^	**.010**	**<.001**	.356	.093	
Δchange	−3.84^**a**^ (−7.02 – 1.43)	−4.52^**a**^ (−9.88 – −1.27)	0.54^**b**^ (−1.27 – 6.95)	1.37^**b**^ (−1.11 – 6.33)	**<.001**

Abbreviations: C, Control group; Cm, *Cornus mas L*. group; DCm, Diet + *Cornus mas L*. group; D, Diet group.

Data are presented as median (25–75 presential).

*p* ^1^ values were calculated using Kruskal–Wallis test; *p* ^2^ values were calculated using Wilcoxon test. There is a statistical difference between the data that do not have a common letter as a superscript.

Table 3 presents the effects of the interventions on biochemical parameters. It also shows the Δchange from the baseline to the end of intervention. As presented, Δchange in fasting insulin, c‐peptide, and HOMA‐IR in DCm was approximately two times greater than in D, and these changes were statistically significant (respectively, between the two groups; *p* = .049; *p* = .020; *p* = .048). Also, it was found that fasting blood glucose, fasting insulin, and HOMA‐IR had a greater Δchange in Cm when compared to C (respectively, between the two groups; *p* = .003, *p* < .001, *p* < .001). No complications or adverse effects were reported by the participants in any of the groups.

**TABLE 3:** Changes in biochemical parameters from baseline to after intervention.

Parameters	DCm (*n* = 22)	D (*n* = 21)	Cm (*n* = 21)	C (*n* = 20)	*p* ^1^
Fasting Blood Glucose (mg/dL)					
Baseline	94.0 (90.0 – 101.5)	97.0 (87.0 – 102.0)	98.0 (93.0 – 104.0)	91.0 (88.0 – 97.0)	.132
After intervention	90.0 (83.8 – 99.0)	93.0 (86.0 – 100.0)	94.0 (89.0 – 98.0)	95.0 (90.0 – 102.8)	.357
*p* ^2^	**.003**	.217	**.003**	.190	
Δchange	−7.67^**a**^ (−9.46 – −0.77)	−4.12^**ab**^ (−10.21 – 5.42)	−6.66^**a**^ (−10.89 – −0.47)	2.07^**b**^ (−3.21 – 10.81)	**.008**
HbA1c (%)					
Baseline	5.6 (5.5 – 5.9)	5.7 (5.6 – 6.1)	5.6 (5.4 – 6.0)	5.6 (5.5 – 6.1)	.443
After intervention	5.4^**a**^ (5.2 – 5.6)	5.5^**bc**^ (5.4 – 5.9)	5.4^**ab**^ (5.3 – 5.6)	5.7^**c**^ (5.4 – 5.9)	**.008**
*p* ^2^	**<.001**	**.027**	**.007**	.530	
Δchange	−3.70^**a**^ (−6.72 – −3.39)	‐3.51^**ab**^ (−6.61 – 0.00)	−1.89^**ab**^ (−6.78 – −0.27)	−1.73^**b**^ (−4.67 – 1.88)	**.046**
Fasting Insulin (uU/mL)					
Baseline	15.6 (12.6 – 18.9)	14.4 (12.2 – 19.5)	16.5 (13.1 – 20.1)	17.8 (12.9 – 22.2)	.685
After intervention	10.2^**a**^ (8.8 – 11.1)	12.1^**b**^ (10.2 – 14.5)	15.0^**c**^ (12.4 – 17.7)	18.8^**d**^ (16.0 – 24.9)	**<.001**
*p* ^2^	**<.001**	**.003**	**.042**	**.003**	
Δchange	−35.90^**a**^ (−48.40 – −15.33)	−16.16^**b**^ (−38.45 – −6,32)	−15.38^**b**^ (−26.69 – 5.77)	15.17^**c**^ (2.98 – 18,29)	**<.001**
C‐peptide (mg/mL)					
Baseline	2.86 (2.59 – 3.12)	2.91 (2.54 – 3.51)	3.06 (2.55 – 3.42)	3.39 (2.64 – 4.04)	.140
After intervention	2.21^**a**^ (2.04 – 2.46)	2.56^**a**^ (1.97 – 2.89)	3.02^**b**^ (2.63 – 3.32)	3.17^**b**^ (2.99 – 4.35)	**<.001**
*p* ^2^	**<.001**	**.002**	.543	.211	
Δchange	−19.49^**a**^ (−27.31 – −12.12)	−9.22^**b**^ (−23.85 – −2.09)	−3.06^**bc**^ (−11.08 – 10.55)	6.56^**c**^ (−7.28 – 18.29)	**<.001**
HOMA‐IR					
Baseline	3.55 (2.91 – 4.67)	3.84 (2.77 – 4.55)	4.14 (3.13–5.25)	4.12 (2.81 – 5.13)	.634
After intervention	2.22^**a**^ (1.86 – 2.63)	2.89^**b**^ (2.22 – 3.48)	3.57^**c**^ (2.83 – 4.06)	4.51^**d**^ (3.72 – 5.84)	**<.001**
*p* ^2^	**<.001**	**.003**	**.013**	**<.001**	
Δchange	−41.07^a^ (−49.53 – −20.79)	−21.17^b^ (−46.59 – −3.54)	−21.34^b^ (−32.60 – 1.85)	21.81^c^ (3.74 – 37.43)	**<.001**
Total Cholesterol (mg/dL)					
Baseline	180.0 (165.8 – 205.0)	172.0 (152.0 – 203.0)	170.0 (146.5 – 201.5)	172.5 (144.3 – 213.5)	.761
After intervention	177.0 (160.0 − 196.0)	165.0 (142.5 – 193.5)	174.0 (146.0 – 96.0)	179.5 (163.3 – 198.3)	.735
*p* ^2^	.626	.118	.945	.130	
Δchange	−1.41^**ab**^ (−7.02 – 5.17)	−3.27^**a**^ (−13.99 – 3.92)	2.34^**ab**^ (−5.73 – 7.21)	3.48^**b**^ (−2.42 – 3.94)	**.033**
LDL‐c (mg/dL)					
Baseline	106.5 (85.8 – 125.5)	100.0 (86.0 – 112.0)	97.0 (63.5 – 123.0)	96.5 (81.0 – 124.5)	.640
After intervention	102.0 (85.0 – 119.5)	98.0 (73.5 – 115.0)	99.0 (81.0 – 111,0)	103.0 (88.3 – 136.0)	.523
*p* ^2^	.372	.350	.668	**.020**	
Δchange	−3.12 (−14.75 – 6.42)	−1.77 (−19.36 – 5.48)	0.00 (−11.13 – 17.45)	6.77 (−3.23 – 18.74)	.070
HDL‐c (mg/dL)					
Baseline	55.5 (47.0 – 61.0)	46.0 (43.5 – 58.0)	50.0 (43.5 – 55.5)	46.5 (36.8 – 61.3)	.170
After intervention	57.5^**a**^ (79.8 – 62.3)	50.0^**ab**^ (45.5 – 61.0)	50.0^**b**^ (46.0 – 55.0)	43.0^**b**^ (41.3 – 54.5)	**.015**
*p* ^2^	.134	.125	.776	.445	
Δchange	2.86 (−2.38 – 10.83)	2.22 (−4.37 – 16.70)	0.00 (−3.89 – 10.53)	−1.53 (−8.65–5.34)	.354
Triglyceride (mg/dL)					
Baseline	97.5 (78.0 – 121.8)	114.0 (84.0 – 177.0)	132.0 (98.5 – 182.0)	96.5 (75.0 – 137.8)	.076
After intervention	89.5 (66.0 – 125.0)	92.0 (78.5 – 125.5)	114.0 (93.5 – 161.5)	100.5 (80.0 – 145.3)	.171
*p* ^2^	.299	**.004**	**.012**	.940	
Δchange	−9.13^**ab**^ (−20.50 – 16.53)	−20.43^**a**^ (−30.42 – −6.68)	−13.58^**ab**^ (−20.12 – −4.48)	2.06^**b**^ (−18.15 – 39.65)	**.038**
CRP (mg/L)					
Baseline	5.02 (2.32 – 6.84)	4.72 (2.54 – 13.85)	5.20 (2.09 – 9.06)	6.04 (3.73 – 11.57)	.674
After intervention	3.94^**a**^ (1.03 – 5.35)	2.72^**a**^ (1.48 – 5.65)	5.19^**ab**^ (1.59 – 6.89)	7.08^**b**^ (3.73 – 11.07)	**.019**
*p* ^2^	**.001**	**.007**	.149	.100	
Δchange	−32.17^**a**^ (−48.15 – −7.84)	−22.56^**a**^ (−75.52 – 4.29)	−12.28^**ab**^ (−29.20 – 15.88)	18.38^**b**^ (−16.48 – 58.53)	**.003**

Abbreviations: C, Control group; Cm, *Cornus mas L*. group; CRP, C‐reactive protein; D, Diet group; DCm, Diet + *Cornus mas L*. group; HbA1c, hemoglobin A1c; HDL‐c, high‐density lipoprotein cholesterol; HOMAIR, Homeostatic Model Assessment of Insulin Resistance; LDL‐c, low‐density lipoprotein cholesterol.

Data are presented as median (25–75 presential).

*p* ^1^ values were calculated using Kruskal–Wallis test; *p* ^2^ values were calculated using Wilcoxon test. There is a statistical difference between the data that do not have a common letter as a superscript.

The change in nutritional intake from baseline to end of the intervention is shown in Table 4. There was no significant difference between the groups at the baseline; however, at the end of the intervention, energy intake and carbohydrate (%) were higher, while dietary fat (%) was lower in the groups that received MNT (*p* < .001). Consuming CM every day, a fruit rich in vitamin C, significantly increased vitamin C intake in groups DCm and Cm (respectively; *p* = .003, *p* < .001).

**TABLE 4:** Changes in nutritional intake from baseline to after intervention.

Parameters	DCm (*n* = 22)	D (*n* = 21)	Cm (*n* = 21)	C (*n* = 20)	*p* ^1^
Energy (kcal/d)					
Baseline	1113.0 (703.4 – 1439.4)	1260.2 (878.3 – 1555.1)	1028.3 (927.4 – 1449.4)	1199.6 (759.5 – 1487.2)	.759
After intervention	1455.1^**a**^ (1369.6 – 1644.9)	1505.0^**a**^ (1404.5 – 1667.2)	1121.2^**b**^ (949.0 – 14700)	1096.9^**b**^ (824.0 – 1497.6)	**<.001**
*p* ^2^	**.017**	**.042**	.357	.654	
Carbohydrate (% energy)					
Baseline	43.5 (35.0 – 52.3)	44.0 (38.0 – 47.5)	41.0 (36.0 – 48.5)	40.5 (35.3 – 49.0)	.989
After intervention	45.5^a^ (43.8 – 50.3)	47.8^a^ (45.2 – 50.9)	40.0^b^ (37.5 – 44.0)	40.5^b^ (36.0 – 46.0)	**<.001**
*p* ^2^	.118	**.048**	.322	.259	
Protein (% energy)					
Baseline	14.0 (13.0 – 19.0)	14.0 (12.0 – 16.5)	17.0 (15.0 – 19.0)	14.0 (13.3 – 17.0)	.066
After intervention	19.0 (18.0 – 20.0)	17.6 (15.5 – 19.0)	18.0 (14.5 – 20.0)	19.0 (16.0 – 21.0)	.071
*p* ^2^	**.033**	**.011**	.822	**.013**	
Dietary Fat (% energy)					
Baseline	43.0 (30.3–51.0)	39.0 (36.5 – 48.5)	43.0 (31.0 – 45.5)	43.0 (36.0 – 49.5)	.754
After intervention	33.5^a^ (30.8 – 38.0)	34.4^a^ (33.0 – 371)	41.0^b^ (39.0 – 44.0)	41.5^b^ (38.3 – 44.0)	**<.001**
*p* ^2^	**.040**	**<.001**	.223	.629	
Fiber (g/d)					
Baseline	15.6 (9.1 – 24.3)	11.9 (10.1 – 19.4)	13.0 (10.0 – 16.8)	13.4 (5.6 – 18.1)	.709
After intervention	18.2^a^ (15.4 – 20.5)	13.5^bc^ (9.0 – 17.0)	18.1^ab^ (12.4 – 21.9)	10.6^c^ (7.6 – 18.1)	**.006**
*p* ^2^	.527	.768	**.027**	.737	
Vitamin C (mg/d)					
Baseline	57.3 (30.6 – 104.9)	50.53 (21.1 – 66.1)	53.0 (43.5 – 65.5)	51.4 (27.3 – 74.7)	.613
After intervention	125.5^a^ (100.9 – 154.9)	54.1^b^ (36.3 – 87.4)	108.2^a^ (95.7 – 149.2)	40.2^b^ (18.6 – 74.2)	**<.001**
*p* ^2^	**.003**	.140	**<.001**	.370	

Abbreviations: C, Control group; Cm, *Cornus mas L*. group; D, Diet group; DCm, Diet + *Cornus mas L*. group.

Data are presented as median (25–75 presential).

*p* ^1^ values were calculated using Kruskal–Wallis test; *p* ^2^ values were calculated using Wilcoxon test. There is a statistical difference between the data that do not have a common letter as a superscript.

## 4 DISCUSSION

These RCT results reveal the effects of both individualized MNT and CM, allowing us to compare the consumption of CM added to MNT over 12 weeks in women with insulin resistance.

It is thought that due to its high anthocyanin content, CM may have positive effects on anthropometric measurements. Several possible mechanisms have been suggested to explain the effects; however, the results of the studies are contradictory (Azzini et al., 2017; Basu et al., 2010; Soltani et al., 2015). In a study where 900 mg of CM extract daily was given without any dietary changes to postmenopausal women, it was found that CM was associated with a decrease in body weight, BMI, and WC compared to the placebo group (Gholamrezayi et al., 2019). Conversely, in another study conducted with non‐alcoholic fatty liver disease (NAFLD) patients, CM extract and a placebo given over 12 weeks were compared; no statistical difference was found for body fat percentage and lean body mass (Yarhosseini et al., 2023). In this study, a statistically significant decrease in anthropometric measurements was found at the end of 12 weeks in the DCm and D, who followed an individualized MNT. When both the anthropometric medians of the two groups and the Δchange were compared, in line with the literature, there was no effect found on CM consumption on body weight, BMI, and body fat mass (%) without an individualized MNT.

Studies have shown that cornelian cherry (*Cornus mas* L.) can reduce fasting blood glucose levels and increase the effect of insulin by inhibiting α‐glucosidase and α‐amylase enzyme activities due to its anthocyanin content (Asgary et al., 2014; Matsui et al., 2001). There are a limited number of clinical studies in the literature with CM and the results on biochemical parameters are conflicting since there are differences in the study groups, region the CM was collected from, and the amount and form given in the studies. In a study, it was shown that giving CM to mice for 10 days had a hypoglycemic effect (Mirbadalzadeh et al., 2010). When 900 mg of CM extract for 6 weeks was given, there was no statistical difference in fasting insulin, HOMA‐IR, triglyceride, total cholesterol, and LDL‐C; however, it was determined that the HDL‐C level increased in the CM group (Gholamrezayi et al., 2019). In a study by Asgary et al. (2013), it was determined that total cholesterol, LDL‐C, HDL‐C, and CRP levels were similar between the CM group and the control group, while triglyceride levels were statistically lower in the CM group. According to a study by Aryaeian et al. (2021) when women who consumed 900 mg of CM extract daily over 8 weeks were compared with the placebo group, CRP levels were found to be statistically lower. A study conducted with T2DM patients found that consuming 600 mg/day anthocyanin for 6 weeks increased fasting insulin levels and decreased HbA1c and triglyceride levels (Soltani et al., 2015). In this study, fasting blood glucose, HbA1c, fasting insulin, C‐peptide, and HOMA‐IR values all decreased in DCm, and the difference with the baseline was statistically significant. At the end of the intervention, the HOMA‐IR and C‐peptide values decreased below 2.5 only in DCm, which is the cut‐off point for the diagnosis of insulin resistance. While insulin resistance parameters in the control group were expected to increase at the end of 12 weeks, the decrease in all insulin resistance‐related parameters except C‐peptide in the Cm group may be related to the increase in insulin sensitivity and glucose uptake into cells by the polyphenols that CM contains. By the same mechanism, the decrease in fasting blood glucose was found to be significant in the DCm, but not in the D. When values between groups and Δchanges of insulin resistance‐related parameters were investigated, it can be considered that the administration of CM alone produces similar changes to MNT. The reason why there was no difference in total cholesterol, LDL‐C, and HDL‐C in the groups consuming CM in this study may be due to that all groups were within the reference range at the baseline. Also, the decrease in CRP levels were found to be statistically significant only in the DCm and D groups. This suggests that the decrease in body weight and body fat percentage had a greater effect on CRP than CM.

The best‐known treatment for insulin resistance is to reduce body weight and body fat mass with an individualized MNT (Solomon et al., 2010; Weickert, 2012). In the Diabetes Prevention Program, it has been shown that a 7% body weight loss with 54% carbohydrate‐containing MNT and accompanying physical activity reduces the progression of T2DM by 58% and results in an improvement in insulin resistance‐related parameters (Kitabchi et al., 2005). When the follow‐up results of 15,428 participants over 25 years were examined for the effect of dietary carbohydrate content on mortality, the group with the lowest risk was found to be those with diets containing 50%–55% carbohydrates (Seidelmann et al., 2018). In a meta‐analysis study, high‐protein (25%–32%) diet plans were compared with low‐protein (15%–20%) diets; high‐protein diets resulted in an average of 2 kg more body weight loss and 0.5% lower HbA1c, thus providing a significant improvement. However, there was no difference in fasting blood glucose, serum lipid profiles, or blood pressure between the groups (Dong et al., 2013). Although different rates of fat intake in the treatment of insulin resistance and prevention of T2DM are reported in the literature, it is known that for general health, the total fat content of the diet should not be above 30%–35% (Muscogiuri et al., 2021; Risérus et al., 2009). Adequate fiber intake is associated with a decrease in all‐cause mortality in individuals with diabetes, and individuals with T2DM risk should have a minimum fiber intake of 14 g/day per 1000 kcal (Evert et al., 2019; He et al., 2010). The fact that vitamins A, C, and E have anti‐inflammatory properties and inflammation has an important place in the mechanism of insulin resistance formation draws attention to supplementing with these vitamins (Reifen, 2002; Sánchez‐Moreno et al., 2004; Södergren et al., 2000). A study conducted with Chinese adults reported that the risk of developing T2DM was less than 5% when 140 mg/day of vitamin C was taken, and high vitamin C intake played a protective role (Zhou et al., 2016). In this study, pre‐intervention patients were trying to lose weight on their own knowledge, since they did not receive any nutrition education, and they restricted their energy, especially carbohydrate intake. Therefore, we demonstrate that these values increase similarly in DCm and D groups after the intervention. Also, the percentage of energy from fat was similar in DCm and D and significantly lower. The high intake of both fiber and vitamin C in the DCm and Cm may be due to the content of CM.

Overall, in the post‐intervention comparison of the groups, the anthropometric measurements and Δchange of the measurements were similar in DCm and D; in addition, their nutritional intake was different from the baseline; in fact, the total energy intake and percentage of carbohydrates was higher, while the biochemical parameters were found to be lower in DCm. When the Δchanges were examined, the fact that fasting insulin decreased by 35.90%, c‐peptide by 19.49%, and HOMA‐IR by 41.07% in DCm and was approximately two times higher than D indicates that CM is effective in insulin resistance‐related parameters. In addition, anthropometric measurements and nutritional intake were similar both before and after the intervention in Cm and C, while the Δchange in fasting blood glucose, fasting insulin, and HOMA‐IR was significantly higher in Cm. This also supports that CM has a positive effect on parameters related to insulin resistance. These findings may be due to some beneficial effects which can be attributed to the flavonoids of CM, including anthocyanins. Cell studies have shown that polyphenols in CM have the ability to control blood glucose levels by inhibiting the enzymes α‐amylase and β‐glucosidase (Matsui et al., 2001; Pinto et al., 2010). Furthermore, polyphenols in red–purple fruits have been shown to inhibit SGLT‐1 and prevent glucose absorption from the intestines (Törrönen et al., 2010). It was found in another study that CM fruit polyphenols can induce insulin receptor phosphorylation and promote glucose uptake by tissues (Zhang et al., 2006).

Considering the studies in the literature and the positive effects of CM given in addition to MNT in women with insulin resistance in our study, it is thought that antioxidant compounds including anthocyanin in CM may be effective in reducing inflammation. Due to this mechanism, CM may be effective in preventing further chronic metabolic diseases as well as in the treatment of insulin resistance.

The strengths of the present study are that it was a randomized controlled trial, and while the effect of CM was investigated alone, it was also investigated as added to MNT. The CM used in this study was given in lyophilized dried fruit form—this form is known to be the method that best preserves the antioxidant content. The amount (20 g) was determined according to the carbohydrate and total anthocyanin content. The limitations of this study are that it was a single‐center study and food consumption records were collected as participants’ self‐reports, therefore they may have reported consuming less than they actually did. Also, the follow‐up period was relatively short, and the participants were only women.

## 5 CONCLUSION

In this study, it has been shown that medical nutrition therapy has positive effects in women with insulin resistance and the addition of 20 g of lyophilized dried CM to the diet can improve insulin resistance and may prevent T2DM. Moreover, consumption of only lyophilized dried CM was found to have similar effects as MNT on insulin‐related biochemical parameters. Therefore, lyophilized dried CM may be effective for the treatment of insulin resistance in premenopausal women. Further studies including male individuals, different age groups, and different metabolic disorders should be conducted.

## AUTHOR CONTRIBUTIONS

**Zehra Margot Celik:** Conceptualization (equal); data curation (equal); formal analysis (equal); investigation (equal); methodology (equal); project administration (equal); resources (equal); visualization (equal); writing – original draft (equal). **Mehmet Sargın:** Conceptualization (equal); methodology (equal); resources (equal); supervision (equal); writing – review and editing (equal). **Havva Gonca Tamer:** Conceptualization (equal); resources (equal); supervision (equal); writing – review and editing (equal). **Fatma Esra Gunes:** Conceptualization (equal); formal analysis (equal); methodology (equal); project administration (equal); supervision (equal); visualization (equal); writing – review and editing (equal).

## FUNDING INFORMATION

This study did not receive any grant from funding agencies in the public, commercial, or not‐for‐profit sectors.

## CONFLICT OF INTEREST STATEMENT

The authors declare that they do not have any conflict of interest.

## ETHICAL STATEMENT

The study was approved by the Marmara University Faculty of Medicine Clinical Research Ethics Committee (09.2018.652). Written informed consent was obtained from all study participants. This study was also registered under Clinical Trial number NCT05292300 (http://www.clinicaltrials.gov).

## Data Availability

The data set used and analyzed for this study is available from the corresponding author upon reasonable request.
